# Immunohistochemical Analysis of LGR5 and TROY Expression in Gastric Carcinogenesis Demonstrates an Inverse Trend

**DOI:** 10.29252/.23.2.107

**Published:** 2019-03

**Authors:** Samaneh Saberi, Abbas Piryaei, Esmat Mirabzadeh, Maryam Esmaeili, Toktam Karimi, Sara Momtaz, Afshin Abdirad, Niloofar Sodeifi, Mohammad Ali Mohagheghi, Hossein Baharvand, Marjan Mohammadi

**Affiliations:** 1 HPGC Research Group, Medical Biotechnology Department, Biotechnology Research Center, Pasteur Institute of Iran, Tehran, Iran;; 2 Department of Biology and Anatomical Sciences, School of Medicine, Shahid Beheshti University of Medical Sciences, Tehran, Iran;; 3 Department of Stem Cells and Developmental Biology, Cell Science Research Center, Royan Institute for Stem Cell Biology and Technology, ACECR, Tehran, Iran;; 4 Department of Molecular Medicine, Biotechnology Research Center, Pasteur Institute of Iran, Tehran, Iran;; 5 Department of Pathology, Cancer Institute, Tehran University of Medical Sciences, Tehran, Iran;; 6 Department of Andrology at Reproductive Biomedicine Research Center, Royan Institute for Reproductive Biomedicine, ACECR, Tehran, Iran;; 7 Cancer Institute, Tehran University of Medical Sciences, Tehran, Iran

**Keywords:** Gastric Cancer, *Helicobacter pylori*, Mice, Stem cells Wnt signaling pathway

## Abstract

**Background:**

Two of the Wnt signaling pathway target genes, tumor necrosis factor receptor family member (TROY) and leucine-rich G-protein coupled receptor (LGR5), are involved in the generation and maintenance of gastrointestinal epithelium. A negative modulatory role has recently been assigned to TROY, in this pathway. Here, we have examined their simultaneous expression in gastric carcinogenesis.

**Methods:**

Tumor and paired adjacent tissues of intestinal-type gastric cancer (GC) patients (n = 30) were evaluated for LGR5 and TROY expression by immunohistochemistry. The combination of the percentage of positively stained cells and the intensity of staining was defined as the composite score and compared between groups. The obtained findings were re-evaluated in a mouse model.

**Results:**

TROY expression in the tumor tissue was significantly lower than that of the adjacent tissue (2.5 ± 0.9 vs. 3.3 ± 0.9, *p* = 0.004), which was coincident with higher LGR5 expression (3.6 ± 1.1 vs. 2.7 ± 0.9, *p* = 0.001). This observation was prominent at stages II/III of GC, leading to a statistically significant mean difference of expression between these two molecules (*p* = 0.005). In the *H. pylori* infected-mouse model, this inverse expression was observed in transition from early (8-16 w) to late (26-50 w) time points, post treatment (*p* = 0.002).

**Conclusion:**

Our data demonstrates an inverse trend between TROY down-regulation and LGR5 up-regulation in GC tumors, as well as in response to *H. pylori* infection in mice. These findings support a potential negative modulatory role for TROY on LGR5 expression.

## INTRODUCTION

Gastric cancer (GC) remains the fifth most common malignancy and the third leading cause of cancer death worldwide^[^^1^^,^^2^^]^, despite its global declining rate. GC is usually diagnosed at later stages of the disease, which is associated with poor prognosis^[^^3^^]^. Therefore, understanding the molecular mechanisms of gastric carcinogenesis is of crucial essence in identifying biomarkers for early screening, diagnosis, and monitoring of disease progression. 

The gastrointestinal (GI) lining is constantly exposed to the harsh luminal contents, including microbial, chemical and mechanical stress inducers^[^^4^^]^. Hence, it is designed to constantly undergo self-renewal^[^^5^^]^. This relentless replenishment of the GI epithelium is owed to a limited number of uniquely designed resident adult stem cells that are programmed to provide the specific architecture and function required by the GI tract^[^^6^^]^. Tissue homeostasis and regeneration of the GI epithelium are strongly governed, in the adult stem cells, by the balance in cell proliferation, cell cycle arrest, and cell migration^[^^7^^]^. These targeted adult stem cells reside in specialized microenvironments, called the stem cell niches, that regulate the frequency and timing of cell-renewal^[^^8^^]^. However, during tumor development, intrinsic and/or extrinsic factors interrupt the normal replenishment process, which leads to dysregulation of the homeostatic balance and uncontrollable cell growth^[^^9^^]^. As a result, stem cells, when driven out of balance, may serve to seed tumor development^[^^10^^,^^11^^]^. 

The Wnt signalling pathway is a critical regulator in the homeostasis of GI tissues^[^^12^^-^^14^^]^. The interaction between its multiple ligands regulates the final strength of the resulting signal, which results in the activation of a subset of transcription factors, controlling the stem cells’ identity and/or differentiation fate^[^^15^^]^. The leucine-rich G-protein coupled receptor (LGR5) has been identified as a target gene of the Wnt signalling pathway, which is owed to its promoter-containing multiple binding sites for the T-cell factor/lymphoid enhancer factor (TCF)/β catenin transcription complex^[^^16^^]^. Cell surface expression of LGR5 (in association with R-spondin) strengthens the Wnt signalling pathway, resulting in a stemness-predominant state^[^^17^^]^, which may potentiate the carcinogenic process by enhancing the cellular regeneration of tumors, such as that of the intestine^[^^18^^,^^19^^]^. Overexpression of LGR5 has been documented in various carcinomas, including hepatocellular^[^^20^^]^, colon and rectum^[^^21^^]^, as well as oesophagus^[^^22^^]^. Another target gene of Wnt pathway is TROY (a member of the TNF-receptor superfamily)^[^^16^^]^, with a dual function, one activating the NFkB signalling pathway^[^^23^^]^, the other forming an inhibitory complex with LGR5 in the cellular membranes, which may modulate the Wnt signalling cascade^[^^16^^]^. Dysregulation of TROY expression has also been reported for several other malignancies such as melanomas^[^^24^^]^, glioblastomas^[^^25^^]^, and squamous cell carcinomas^[^^26^^]^. Recent studies on the function of TROY in GI cancers have mostly focused on colorectal cancer^[^^16^^,^^23^^,^^27^^]^, in which the role of Wnt signalling differs from that of gastric tissue. Fafilek and colleagues^[^^16^^]^, having carried out chromatin co-percipitation and DNA microarray studies on various colorectal cancer cell lines, demonstrated the co-precipitation of TROY and LGR5. Additional siRNA studies led them to conclude that TROY may act as a negative modulator of the Wnt signalling pathway, in LGR5-positive stem cells^[^^16^^]^. The function of TROY in GC is poorly understood. Nevertheless, Wilhelm *et al.*^[^^28^^]^ evaluated the expression of TROY in GC patients by immunohistochemistry and qRT-PCR and demonstrated its loss of expression in tumor tissue, as well as loss of colony formation ability in a TROY-over-expressing GC cell line.

In the present study, we aimed to evaluate the proposed negative modulatory role of TROY on LGR5 expression, by studying their simultaneous expression in the neoplastic and non-tumoral adjacent tissues of GC patients, at various stages of disease. We, then, assessed this incident at late versus early time points, following *H. pylori* infection, in a mouse model. Collectively, our data manifests the up-regulation of LGR5 in gastric carcinogenesis, coincident with down-regulation of TROY. 

## MATERIALS AND METHODS

**Patients**

Our study population included 30 patients with histologically confirmed intestinal GC admitted at Cancer Institute, Tehran University of Medical Sciences, Iran. Data and sample collection was performed following the provision of written informed consent, according to the protocols approved by the National Committee on Ethical Issues in Medical Research, Ministry of Health and Medical Education of Iran; Ref No. 315. Patient identifiers were kept confidential. Demographic information, including age, gender, ethnicity, family history of GC, and smoking habits were obtained via personal interviews (Table 1). Gastric tissue from the tumor (neoplastic) and the corresponding adjacent (non-tumoral) regions were obtained following partial or total gastrectomy. 

**Serologic assays**

Fasting venous blood was collected from each subject, prior to gastric surgery. The sera were isolated and stored at -70 °C until later measurement of anti-*H. pylori* IgG and serum pepsinogen I (sPGI) and II (sPGII) levels. 

**Table 1:** Demographic and clinicopathological characteristics of the study population

**Characteristics **	**n (%)**
Age (y)	
Mean ± SD	67.4 ± 5.72
Median	66.5
Range (min, max)	25 (54, 79)
Gender	
Female	10 (33.3)
Male	20 (66.7)
Ethnicity	
Fars	6 (20.0)
Non-Fars	24 (80.0)
Family history of GC	
No	19 (63.3)
Yes	11 (36.7)
Smoking habits	
Never	20 (66.7)
Ever	10 (33.3)
*H. pylori*	
Sero-negative	6 (20.0)
Sero-positive	24 (80.0)
PGI/II ratio	
>3.0	14 (46.7)
≤3.0	16 (53.3)
Subsite	
Proximal	19 (63.3)
Distal	11 (36.7)
Grade	
Well	11 (36.7)
Moderate	9 (30.0)
Poor	10 (33.3)
Stage	
I	5 (16.7)
II	5 (16.7)
III	17 (56.7)
IV	3 (9.9)

***H. pylori sero-status***

The presence of serum IgG against *H. pylori *was detected as previously described^[^^29^^]^. Subjects with borderline results were re-evaluated by a commercial ELISA kit (Trinity Biotech, Ireland).

**Serum pepsinogens**

sPGI and sPGII levels were measured by ELISA (Biohit, Finland) according to the manufacturer’s instructions, and the PGI/II ratio was calculated. Serum PGI/II values were dichotomized based on the proposed cut-off value of 3.0^[^^30^^,^^31^^]^. 

**Clinicopathologic features**

Patients were classified according to the subsite as proximal or distal. Paraffin-embedded gastric tissues were sectioned, stained with H & E stain and evaluated by an expert pathologist. Gastric tumors were categorized according to their differentiation grade (well/moderate/poor)^[^^32^^]^ and stage of progression (I, II, III, IV) according to TNM classification (T: primary tumor, N: regional lymph nodes, M: distant metastasis)^[^^33^^]^.

**Bacteria**

Mouse-adapted *H. pylori* strain was grown at 37 °C under micro-aerobic conditions (10% CO_2_, 5% O_2_, and 85% N_2_) up to 72 h. The bacteria were then harvested from solid culture, suspended in Brucella Broth and incubated at 37 °C, while shaking at 150 rpm, up to 24 hours^[^^34^^]^. 

**Mouse treatment**

Six-week-old female C57BL/6 mice (n = 54) were procured from Pasteur Institute of Iran (Tehran) and housed at the animal care facilities, in accordance with approved standards. The mice were treated as previously described for gerbils^[35]^. Briefly, the animals were randomized into treatment (n = 26) and sham (n = 28) groups. The former group was given 200 ppm MNU (N-methyl-N-nitrosourea, Sigma, USA) dissolved in their drinking water, for one week. They were then orally gavaged with *H. pylori* inoculums (10^8^ cfu dissolved in 200 μl of Brucella Broth) thrice, with one-day intervals. They were also fed high (10%)-salt diets, with weekly intervals, for 50 weeks thereafter. The sham-treated mice received 200 μl of Brucella Broth, instead of *H. pylori* inoculums and were fed a regular diet. Gastric tissues were collected from both groups, following euthanization by intraperitoneal injection of overdose of ketamine and xylazine (Alfasan, Woerden, Holland) at 8, 16, 26, and 50 weeks post treatment. Paraffin-embedded gastric tissues of mice were sectioned, stained with H&E stain and evaluated by an expert pathologist. Data obtained at early (8 + 16 w) and late (26 + 50 w) time points were pooled, for the sake of statistical analysis. 

**Immunohistochemistry (IHC)**

Formalin-fixed and paraffin-embedded tissues were sectioned (5 μm thickness), mounted on poly-L-lysine coated slides, deparaffinized in xylene and rehydrated. Endogenous peroxidase was blocked by exposure to 3% H_2_O_2_ for 10 min, followed by antigen retrieval *via* pressurized heating in the Dako retrieval buffer (Dako, Denmark) for 1 hour. After cooling to room temperature, non-specific sites were blocked by exposure to 10% goat blood serum. LGR5 and TROY immune staining was performed using a rabbit polyclonal anti-LGR5 (dilution 1:400, ab75732, Abcam Inc., Cambridge, USA) and rabbit monoclonal anti-TROY antibody (dilution 1:600, ab138502, Abcam Inc., Cambridge, USA), respectively. Incubation with the primary antibody was done in a moist chamber at 4 °C. For visualization, slides were sequentially incubated with horse radish peroxidase-conjugated anti-rabbit IgG (dilution 1:1000, ab205718, Abcam Inc., Cambridge, UK) and DAB substrate kit (Dako, Denmark). The nuclei were counterstained with hematoxylin. 

**Evaluation of immunostaining**

The processed immunostained sections were examined by our expert histologist, under light microscopy, in a blinded fashion. LGR5+ and TROY+ cells were scored as previously described^[^^36^^]^. Briefly, the two variables of (a) percentage of positively stained cells (0 [none], 1 [1-25%], 2 [25-50%], 3 [>50%:]) and (b) the intensity of cytoplasmic staining (0 [no staining], 1 [mild], 2 [moderate], 3 [strong], with the highest intensity score being assigned when >10% of cells stained with that intensity] were combined to create the composite score (CS = 0-6). 

**Statistical analysis**

Statistical analyses were done using SPSS 20.0 (IBM Corporation, New York, USA) and Graphpad Prism 6 (Graphpad Software, Inc., La Jolla, USA). The differences in mean CS of LGR5 and TROY expression between different groups were analyzed by Spearman’s rank correlation analysis. The association between LGR5 and TROY expression and clinicopathological characteristics was evaluated by Student's *t*-test. Grouped histochemical data were analyzed using one-way ANOVA. A two-sided *p *value < 0.05 was considered as statistically significant. 

## RESULTS

**Patient composition**

Histologically confirmed intestinal type GC patients were included in this study. The demographic and clinicopathologic characteristics of the studied population are listed in Table 1. Briefly, the studied patients were of the median age of 66.5 years, the majority of whom were of the male gender (66.7%) and non-Fars ethnicity (80%), with no family history of GC (63.3%). Most of these patients were never-smokers (66.7%). Their gastric tumors were mostly located in the proximal region of the stomach (63%), with varying grades of differentiation and stages of progression. *H. pylori*-seropositive subjects (80%) predominated the study population, and patients were similarly distributed among both categories of PGI/II.

**Qualitative expression of LGR5 and TROY in gastric cancer patients**

**Fig. 1:**
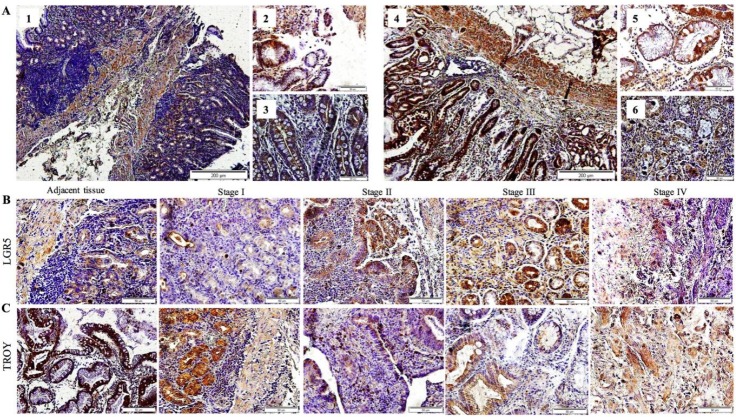
Immunohistochemical detection of LGR5 and TROY expression in the gastric tissue of gastric cancer patients. Overview of LGR5+ (1-3) and TROY+ (4-6) cells in (A) non-tumoral adjacent tissue. Scale bars of 1 and 4, 200 µM; 2, 3, 5, and 6, 50 µM. Representative slides of (B) LGR5 and (C) TROY immunostaining in transition from non-tumoral adjacent tissue to that of tumor, progressing from stage I to IV (scale bars 50 µM)

LGR5 expression in the non-tumoral adjacent tissue of GC patients was mainly localized to the gastric epithelial cells in the basal portion of the glands (Fig. 1 A: 1), with higher intensity in the cell membranes (Fig. 1A: 2) as opposed to their cytoplasms (Fig. 1A: 3). Conversely, TROY was broadly expressed throughout the gastric epithelial layer of the non-tumoral adjacent tissue, similarly distributed amongst the glands and surface epithelium (Fig. 1A: 4), in the cell membranes as well as their cytoplasm (Fig. 1A: 5 and 6). Taken together, a larger proportion of morphologically differentiated cells in the gastric epithelium of the non-tumoral adjacent tissue of GC patients expressed TROY rather than LGR5. 

**Quantitative expression of LGR5 and TROY in gastric tumors and non-tumoral adjacent tissues**

**Fig. 2:**
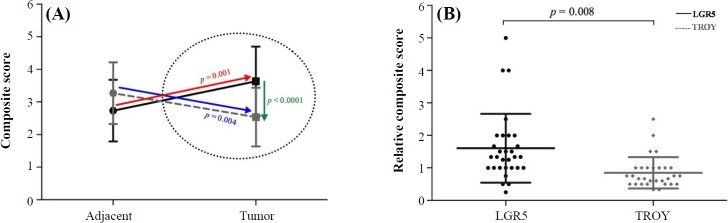
Expression of LGR5 and TROY mean composite scores (CS) of (A) tumor and non-tumoral adjacent tissues and (B) relative expression (CS_tumor_/CS_adjacent_) of LGR5 and TROY. The arrows in panel A depict the inverse trend

In this study, the immune-stained sections were evaluated and scored, based on the percentage of positively stained cells, in combination with the cytoplasmic staining intensity and reported as the composite score (CS). More detailed analysis of the CS of non-tumoral (adjacent) tissues of patients *versus* their tumoral tissue revealed down-regulation of TROY expression (from 3.3 ± 0.9 to 2.5 ± 0.9, *p* = 0.004, Fig. 2A), coinciding with up-regulation of LGR5 expression (from 2.7 ± 0.9 to 3.6 ± 1.1, *p* = 0.001, Fig. 2A), which resulted in a highly significant mean difference of the two molecules in the tumor tissue (*p* < 0.0001, Fig. 2A). Furthermore, we have defined a novel variable, by which the tumoral expression of the molecule of interest was normalized against their paired adjacent (non-tumoral) counterparts (tumor CS/adjacent CS), and referred to as the relative CS (RCS). Accordingly, the mean RCS of LGR5 (1.6 ± 1.1) was significantly higher than that of TROY (1.1 ± 0.6, *p* = 0.008, Fig. 2B). 

**The association of gastric tissue expression of LGR5 and TROY with patients’ demographic and clinicopathologic characteristics**

Pearson correlation analysis demonstrated increasing expression of TROY in the tumor-adjacent normal tissue, with rising age (r = 0.362, *p* = 0.049, Table 2), which did not hold true for that of the tumor tissue. In patients with family history of GC, significantly higher tumoral expression of LGR5 was observed (mean CS = 4.5 ± 0.5), in reference to those without (mean CS = 3.1 ± 0.9, *p* < 0.0001, Table 2). However, this result was not observed for TROY expression. Besides, no statistically significant association was observed with gender or ethnicity of the patients, or their smoking habits and expression of either of these molecules (Table 2). 

Tumoral expression of LGR5 was significantly higher in *H. pylori* sero-positive patients (3.9 ± 0.9), in reference to sero-negative ones (2.7 ± 1.4, *p* = 0.01, Table 3). Accordingly, those with serum PGI/II ratio of ≤3.0 had higher levels of tumor LGR5 expression (3.9 ± 1.1), as compared to those above 3.0 (3.3 ± 1.0), which did not reach statistical significance (*p* = 0.095, Table 3). The contrary occurred for TROY, which demonstrated significantly lower levels of tumor expression, in those with serum PGI/II ratio of ≤3.0 2.6 ± 1.1), but not in those above 3.0 (3.5 ± 1.2, *p* = 0.035, Table 3).

Based on the anatomic location of the tumors, those in the distal stomach had a higher expression of LGR5 (4.2 ± 0.8), in reference to those in the proximal region (3.3 ± 1.1, *p* = 0.029, Table 3). On the other hand, TROY expression was higher in the tumor-adjacent normal tissue of the distal stomach (3.5 ± 1.1) versus the proximal (2.7 ± 0.8) region, which was borderline significant (*p* = 0.052, Table 3). 

**Table 2:** The association of LGR5 and TROY expression with patients’ demographic characteristics

**Demographic ** **characteristics**	**Total** **(n)**	**LGR5 ** **Mean ± SD**			**TROY ** **Mean ± SD**		
		**ACS**		**TCS**	**ACS**		**TCS**
Age (correlation)							
*p* value		0.915		0.189	0.049		0.773
Gender							
Female	10	2.8 ± 1.1		3.7 ± 1.3	3.2 ± 0.9		3.0 ± 1.5
Male	20	2.9 ± 1.0		3.6 ± 1.0	2.9 ± 1.0		3.1 ± 1.1
*p *value		0.809		0.813	0.440		0.838
Ethnicity							
Fars	6	2.3 ± 0.8		3.7 ± 0.5	3.5 ± 0.8		3.8 ± 1.0
Non-Fars	24	3.0 ± 1.1		3.6 ± 1.2	2.9 ± 1.0		2.9 ± 1.2
*p *value		0.165		0.934	0.167		0.088
Family history of GC							
No	19	2.8 ± 1.0		3.1 ± 0.9	3.2 ± 1.0		3.2 ± 1.2
Yes	11	3.0 ± 1.2		4.5 ± 0.5	2.7 ± 0.9		2.8 ± 1.3
*p* value		0.603		<0.0001	0.254		0.409
Smoking Habits							
Never	20	2.8 ± 1.0		3.7 ± 1.0	3.1 ± 0.7		3.1 ± 1.4
Ever	10	3.1 ± 1.1		3.6 ± 1.2	2.8 ± 1.4		3.1 ± 0.9
*p value*		0.395		0.906	0.440		0.919

ACS, adjacent composite score; TCS, tumor composite score

**Fig. 3:**
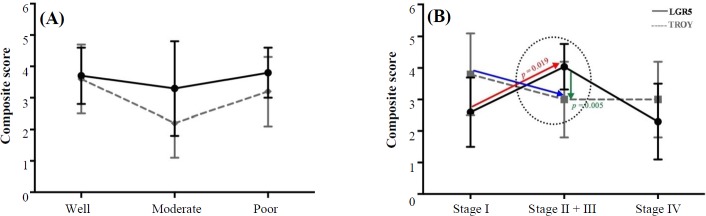
Expression of LGR5 and TROY at (A) different grades of tumor differentiation and (B) stages of disease progression. The arrows in panel B depict the inverse trend

With regard to tumor progression, one-way ANOVA analysis identified a statistically significant association between the grade and stage of disease with TROY (*p* = 0.028) and LGR5 (*p* < 0.0001) expression, respectively (Table 3). A more detailed two-by-two analysis demonstrated a statistically significant down-regulation of TROY expression, as the tumors lost differentiation (well to moderate, *p* = 0.009, Fig. 3A). Of higher interest was the observation of TROY down-regulation in transition from stage I to III, simultaneous with statistically significant up-regulation of LGR5 expression (*p* = 0.019), leading to a statistically significant mean difference of expression between these two molecules at stages II and III (*p* = 0.005). This finding was also evident in representative slides of LGR5 and TROY immunostaining in transition from non-tumoral adjacent tissue to that of tumor, progressing from stage I to IV. In the course of tumorigenesis, up to stage III, an overall up-regulation of LGR5 was exhibited, simultaneous with down-regulation of TROY, (Fig. 1B and 1C). At stage IV of the disease, loss of cellular differentiation was observed. These features include organelle shrinkage and polarity loss, as well as affected plasma membrane integrity, which presented a fibroblastic-like morphology (Fig. 1B and 1C, stage IV). 

**Gastric tissue expression of LGR5 and TROY in mice**

**Fig. 4:**
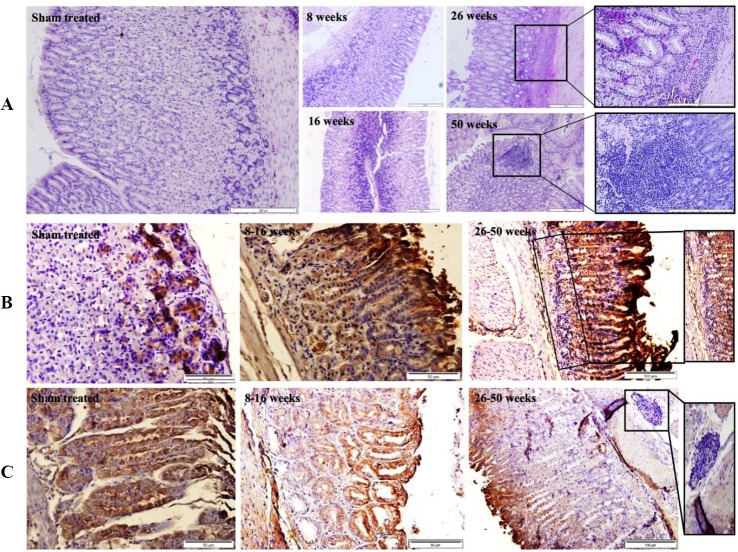
Representative slides of mouse gastric tissue. (A) H&E staining (scale bars: 200 µM), (B) immunodetection of LGR5 (scale bars: 50 µM), and (C) TROY (scale bars: 50 µM) expression at different time points post treatment

**Fig. 5:**
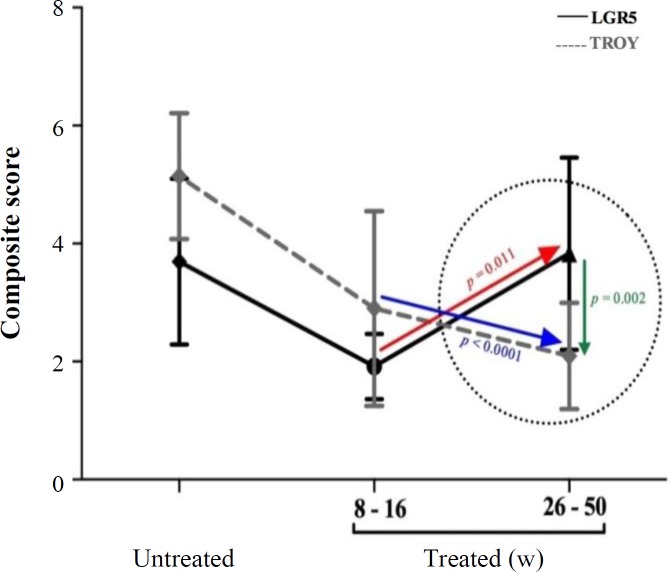
Expression of LGR5 and TROY in mice during weeks post treatment. The arrows demonstrate the inverse trend

In light of the association of LGR5 expression with *H. pylori* seropositivity, we evaluated the gastric tissue expressions of LGR5 and TROY, in a mouse model, during early and late time points following *H. pylori* infection. Microscopic examination of the H&E slides demonstrated infiltration and accumulation of mononuclear cells at the lamina propria of the gastric mucosa as early as eight weeks post treatment (Fig. 4A2), which accelerated through time and yielded the formation of structurally organized lymphoid follicles at 50 weeks post treatment (Fig. 4A5). In transition from early (8-16 w) to late (26-50 w) time points post treatment, the mentioned inverse trend, which was observed in GC patients, was also observed in the treated mice (Fig. 5). Such that TROY expression was down-regulated in mice, in transition from early (8-16 w) to late (26-50 w) time points post treatment (*p* <0.0001, Fig. 5), simultaneous with up-regulation of LGR5 expression of (*p* = 0.011, Fig. 5), leading to a statistically significant mean difference of composite scores between these two molecules at late (26-50 w) time points (*p* = 0.002, Fig. 5). This phenomenon can be observed in the representative slides of LGR5 (Panel B) and TROY (Panel C) immunostaining in transition from sham-treated state (Fig. 4B1 and 4C1), to early time points (Fig. 4B2 and 4C2) and progressing to late time points (Fig. 4B3 and 4C3). In the course of disease progression in mice, an overall up-regulation of LGR5 was exhibited, simultaneous with down-regulation of TROY, (Fig. 4B and 4C). Localization of these two molecules in the mouse gastric mucosa was similar to that of humans.

**Table 3:** The association of LGR5 and TROY expression with clinicopathological characteristics

**Demographic ** **Characteristics**	**Total** **(n)**		**LGR5 ** **Mean ± SD**					**TROY ** **Mean ± SD**		
			**ACS**		**TCS**			**ACS**		**TCS**
*H. pylori*										
Sero-negative	6		2.5 ± 1.0		2.7 ± 1.4			3.2 ± 0.8		3.2 ± 1.2
Sero-positive	24		3.0 ± 1.0		3.9 ± 0.9			3.0 ± 1.0		3.0 ± 1.3
*p *value			0.344		0.010			0.650		0.828
PGI/II ratio										
>3.0	14		2.7 ± 1.2		3.3 ± 1.0			3.1 ± 1.2		3.5 ± 1.2
≤3.0	16		3.0 ± 0.9		3.9 ± 1.1			2.9 ± 0.8		2.6 ± 1.1
*p *value			0.463		0.095			0.467		0.035
Subsite										
Proximal	19		3.0 ± 1.1		3.3 ± 1.1			2.7 ± 0.8		2.9 ± 1.1
Distal	11		2.6 ± 1.0		4.2 ± 0.8			3.5 ± 1.1		3.4 ± 1.4
*p *value			0.366		0.029			0.052		0.323
Grade										
Well	11		2.6 ± 1.3		3.7 ± 0.9			2.9 ± 1.0		3.6 ± 1.1
Moderate	9		3.1 ± 1.1		3.3 ± 1.5			2.9 ± 1.1		2.2 ± 1.1
Poor	10		2.9 ± 0.7		3.8 ± 0.8			3.2 ± 0.9		3.2 ± 1.1
*p *value			0.840		0.610			0.746		0.028
Stage										
I	5		3.2 ± 1.3		2.6 ± 1.1			3.6 ± 0.9		3.8 ± 1.3
II	5		3.0 ± 0.7		4.6 ± 0.5			3.2 ± 0.4		3.2 ± 1.5
III	17		2.8 ± 1.1		3.8 ± 0.7			2.8 ± 1.1		2.9 ± 1.0
IV	3		2.6 ± 1.2		2.3 ± 1.2			3.0 ± 1.0		3.0 ± 1.2
*p *value			0.323		<0.0001			0.410		0.396

ACS, adjacent composite score; TCS, tumor composite score

## DISCUSSION

There is limited information on the interaction of LGR5 and TROY in the process of gastric carcinogenesis. Our simultaneous assessment of these two molecules in GC patients, by IHC, demonstrated that the up-regulation of LGR5 coincides with the down-regulation of TROY in the tumor tissue. Previous pertinent IHC studies on these two molecules, in GC are summarized in Table 4. Every previous study that has performed paired assessment of LGR5 expression in GC tumor versus its non-tumoral adjacent tissue has reported the up-regulation of LGR5 in the former tissue (Table 4)^[^^36^^-^^39^^]^. In reference to immune detection of TROY expression, there is only one report by Wilhelm and colleagues^[^^28^^]^ who have demonstrated its down-regulation in gastric tumor, as compared to their paired non-tumoral counterparts (Table 4). To our knowledge, we are the first to assess the simultaneous expression of LGR5 and TROY in GC. Regarding to the clinic-pathologic features, patients with family history of GC, a risk factor for predisposition to GC^[^^40^^]^, manifested higher tumoral expression of LGR5. It is well known that a family history of GC potentiates the risk for developing this type of cancer, reviewed by Yusefi *et al.*^[^^41^^]^. In this study, we have observed that these subjects have higher tumoral expression of LGR5, which may indicate a pro-carcinogenic status. However, this hypothesis can be validated only if demonstrated in a prospective study, prior to tumor development. Of particular interest is the fact that even though all our studied patients were diagnosed with confirmed GC, amongst them, those who are seropositive for *H. pylori* infection demonstrated higher tumoral expression of LGR5. In accordance with our findings, Uehara *et al*.^[^^42^^]^ and Choi *et al*.^[^^43^^]^ have denoted increased expression of LGR5 in gastric epithelial cells of *H. pylori*-positive GC patients (Table 4). Concordant with *H. pylori*-induced atrophic changes of gastric secretory glands, the level of serum PG declines^[^^44^^,^^45^^]^, such that low serum PGI/II ratio is a serum risk indicator for GC^[^^31^^,^^46^^]^. In our study, GC patients, with low serum PGI/II ratio, as opposed to those above, expressed higher levels of LGR5 and lower levels of TROY, in their tumor tissues. With regard to the differentiation grade of GC, we found decreased TROY expression, as the tumor lost differentiation, in transition from well to moderate grade, which was not detectable in poorly differentiated tumors. In agreement with our findings, in moderately differentiated tumors, Wilhelm *et al*.^[^^28^^]^ observed a correlation of TROY expression with loss of tumor differentiation. Meanwhile, LGR5 expression has repeatedly been demonstrated to be associated with the tumor grade of differentiation^[^^36^^,^^38^^,^^39^^,^^47^^-^^49^^]^, with differing orders. On the other hand, the stage of tumor is a commonly used index to evaluate disease progression. Here, we have observed the downward trend of TROY expression in transition from stage I to III, simultaneous with up-regulation of LGR5 expression, manifesting a clear inverse trend. The former observation for TROY has been supported by a recent study^[^^28^^]^, which reported a declining trend of its expression as tumor stage progresses. In reference to accelerating levels of LGR5, literature is divided into two categories. One that supports our findings of a direct association^[^^37^^,^^39^^,^^48^^,^^50^^,^^51^^]^ with tumor stage progression, and the other that reports the reverse^[^^36^^,^^38^^]^. Our observation of elevated levels of LGR5 up to stage III and its decline thereafter has been denoted by others as well^[^^37^^,^^50^^,^^51^^]^. Nevertheless, both groups confirm a distinctive association between tumoral expression of LGR5 and tumor stage (Table 4). These results candidate both LGR5 and TROY, not only as diagnostic, but also as prognostic markers, especially since their levels correlate with the survival rate of the patients (Table 4)^[^^28^^,^^37^^-^^39^^,^^48^^,^^50^^,^^51^^]^. 

**Table 4:** Review of immunohistochemistry studies on LGR5 and TROY expression in gastric cancer

**Country/** **year**		**Study population**							**Results**							**Application**	
		**Cases**	**Controls**			**Inclusion** **criteria**			**Staining** **pattern**	**Cases vs.** **controls**	**Demographic characteristics**	**Clinico-pathological** **characteristics**		**Survival**		**Diagnostic**	**Prognostic**
**LGR5**																	
Iran 2019^[This study]^		GC (n = 30)	Paired adjacent			Intestinal GC			M/C	GC > adjacent	Family history of GC (yes > no)	Hp^+^ > Hp^ -^ Tumor subsite (proximal > distal)		-		√	√
													
USA 2018^[^^60^^]^		IM (n = 17) Dys (n = 10) GC (n = 26)	NM (n = 10)			Hp-positive only			M	GC > Dys >IM >NM	Male > female	Tumor stage (II/III > I/IV)		-		√	√
													
Iran 2017^[^^49^^]^		GC (n = 94)	Case only			No previous treatment			M/C	Case only	> 63 y > ≤63 y	Tumor subtype (intestinal > diffuse) Tumor grade (moderate > well > poor)		-		-	√
													
Colombia 2017^[^^61^^]^		IM (n = 17) Dys (n = 10) GC (n = 26)	NM (n = 10)			Hp-positive only			-	GC > Dys > IM > NM	-	-		-		√	√
													
China 2017^[^^62^^]^		IM (n = 16)	Paired adjacent			IM (lesser curvature only)			M	IM < adjacent	-	IM grade (3 < 2 < 1 < 0)		-		√	√
													
Korea 2016^[^^43^^]^		Dys (n = 21) Early GC (n = 25)	NM (n = 30)			Current Hp infection Early GC			-	GC > Dys > NM	Current Hp infection > Hp eradicated Hp^+^ NM> Hp^-^ NM Hp^+^ GC > Hp^-^ GC	Antrum > body Dys (moderate > severe > mild > none)		-		√	√
													
China 2016 ^[^^51^^]^		PL of GC (n = 377) Metastatic LNs of GC (n = 194)	Adjacent (n = 93)			-			C	-	(> 60 y) > (45-60 y) > (≤45 y) Male > female	Tumor invasion (no > yes) Tumor stage (III > IV > I-II)				√	√
													
China 2015^[^^39^^]^		GC (n = 261)	Paired adjacent ( n = 261)			-			M/C	GC > adjacent	NS	Tumor size (> 8 > 4-8 < > 4) Tumor grade (poor > moderate > well) Tumor stage (III-IV > I-II)				√	√
													
China 2014^[^^48^^]^		GC (n = 318)	NM (n = 80)			-			C	GC > adjacent	NS	Tumor grade (poor > moderate > well) Tumor stage (IV > III > II > I)				√	√
													
China 2014^[^^50^^]^		GC (n = 68)	Case only			Preoperative (oxaliplatin-based) chemotherapy			C	-	NS	Tumor size (>8 > 4-8 > <4) Tumor grade (poor > moderate/well) Tumor stage (III > I-II)				-	√
													
USA 2014^[^^63^^]^		Hp^+^ Gastritis (n = 12) Hp^+^IM (n = 10) Dys (n = 6) GC (n = 12)			Hp^-^ M (n = 10)	-			-	GC/IM > NM	-	NM (Hp+ > Hp-)		-		-	√
													
China 2013^[^^38^^]^		GC (n = 236)	Paired adjacent (n = 236)			-			M/C	GC > adjacent	>60 y > ≤60 y Male > female	Subtype (intestinal > mixed > diffuse) Tumor grade (well/moderate > poor) Tumor stage (I-II > III-IV)				√	√
													
China 2013^[^^47^^]^		GC (n = 160)	NM (n = 99)			-			-	-	-	Tumor differentiation (well > moderate > poor) Tumor subsite (proximal > distal)		-		√	√
													
China 2013^[^^36^^]^		IM (n = 90) Dys (n = 53) GC (n = 180) GC with metastases in lymph nodes and the liver (n = 15)		Paired adjacent (n = 145)		-			M/C	All cases > adjacent IM > adjacent Dys with IM (yes > no)	>60 y > ≤60 y Male > female	Tumor Subtype (intestinal > diffuse) Dys (low > high) Tumor grade (differentiated > undifferentiated) Tumor stage (I-II > III-IV) Metastasis or recurrence (No > Yes)		-		√	√
													
USA 2013^[^^42^^]^		GC (n = 35)	Non-GC (n = 18)			-			C	-	All (Hp^+^ > Hp^-^) GC (Hp^+^ > Hp^-^) Non-GC (Hp^+^ > HP^-^)		Antrum > oxyntic	-		-	√
													
Germany 2012^[^^37^^]^		GC (n = 127)	Paired adjacent (n = 127)			Intestinal GC			M/C	Tumor > adjacent	≥71 y > <71 y	Tumor stage (III > IV > II > I)				√	√
													
													
**TROY**																	
Iran 2019^[This study]^		GC (n = 30)	Paired adjacent			Intestinal GC			M/C	GC < adjacent	Age (positive correlation)	PG (> 3.0) > (≤ 3.0) Tumor grade (well >moderate/poor)		-		√	√
													
Germany 2016^[^^28^^]^		GC (n = 52)	Paired adjacent (n = 52) Normal stomach (n = 5)				-		M	GC < Adjacent	-	Tumor grade (inverse correlation) Tumor type (intestinal > diffuse) Tumor stage (I >II >III > IV)				√	√

IHC, immunohistochemistry; Dys, gastric dysplasia; GC, gastric cancer; IM, intestinal metaplasia; LNs, lymph nodes; NM, normal mucosa; NS, not significant; PL, primary lesions; y, years;  , increased; , decreased, M, membrane; C, cytoplasm; M/C, membrane/cytoplasm; Hp, *H. pylori*

In order to evaluate the above-mentioned inverse trend of LGR5 and TROY expression following *H. pylori* infection, we investigated their gastric tissue expression in a mouse model, based on a previous study on cancer in gerbils^[^^35^^]^. Having done so, we again observed a highly significant down-regulation of TROY, in transition from early (8-16 w) to late (26-50 w) time points, which coincided with the up-regulation of LGR5, during the same time period. This elevation occurred in a precancerous state, where *H. pylori*, in the presence of co-carcinogens, may trigger the gastric carcinogenic process. Our latter observation is in agreement with the evidence provided by an eloquent lineage tracing study^[^^52^^]^, which demonstrated that *H. pylori* colonization, of mouse gastric stem cell compartment, affects their turn-over kinetics, resulting in expansion of LGR5-positive stem cell population and up-regulation of stem cell-related genes. This incident may explain our observation of increased tumoral expression of LGR5 in *H. pylori*-positive GC patients. Moreover, it has been documented that certain populations of *H. pylori* have a tropism for the progenitor cell niche^[^^52^^,^^53^^]^. These investigators^[^^52^^,^^53^^]^, have associated this function of *H. pylori* to its virulent capacity, in particular to CagA, which is thought to be responsible for the inhibition of tumor suppression^[^^54^^]^, alteration of cell polarity^[^^55^^]^, and/or deregulation of cellular signalling pathways^[^^56^^]^. To our knowledge, there is no equivalent study for the interaction between *H. pylori* and TROY expression in the mouse stomach. Thus, our study provides the first clue as to its potential negative modulatory role on the expression of LGR5 in mice. 

As previously mentioned, TROY is identified as one of the Wnt target genes, which due to its promoter containing multiple consensus TCF-binding sites, was precipitated with the TCF4-specific antibody^[^^16^^]^. On the other hand, treatment of HEK293 cells with TROY siRNA increases phosphorylation and cellular levels of the Wnt coreceptor, lipoprotein receptor-related protein (LRP) 6, vouching for its negative regulation of Wnt signalling pathway, by reducing LRP phosphor-rylation^[^^16^^]^. In the absence of TROY, increased levels of LRP phosphorylation potentiate the Wnt signaling cascade, leading to the accumulation of beta-catenin and expression of Wnt signalling target genes, including LGR5^[^^16^^]^. Additional support for the negative modulatory role of TROY on the Wnt signalling pathway has been provided by Wilhelm *et al*.^[^^28^^]^, who have demonstrated that the overexpression of TROY in MKN45 cells, a GC cell line, leads to decreased clonal expansion and increased differentiation. In this regard, Wu *et al.*^[^^57^^]^ and Qiu *et al.*^[^^58^^]^ have suggested that the expression of TROY is associated with cellular differentiation in osteoblastogenesis and adipogeneisis. These reports, in addition to our observations, allow us to speculate that in response to carcinogenic stimuli, such as *H. pylori* colonization, once the negative modulation of TROY on the Wnt signalling pathway is lifted, its downstream genes including LGR5 may be over-expressed. This inverse trend may depict a process of cellular dedifferentiation and stem cell expansion potentiating malignant transformation of cells. An indicator of cellular deformation and dedifferentiation can be the decline in the level of their secretory products, i.e. gastric PGIs^[^^44^^]^. Further TROY down-regulation and LGR5 up-regulation in our GC patients, with low serum PGI/II levels, strengthen this hypothesis. Our observations indicate that this process is maximized at stages II and III, after which the LGR5 expression returns to its previous levels. The latter stage may reflect a time point (stage IV) at which dedifferentiated cells begin to gain a disparate differentiation fate^[^^59^^]^, i.e. gastric to intestinal transdifferentiation. According to these speculations, TROY and LGR5 expression levels may reflect differentiated versus dedifferentiated cellular states, respectively. This hypothesis demands further studies for assessing the expression levels of these two molecules, as GC screening markers. 

Collectively, we have observed an inverse trend between TROY down-regulation and LGR5 up-regulation in GC in humans, as well as in response to *H. pylori* infection in mice. These findings provide further evidence for the negative modulatory impact of TROY on the Wnt signalling target genes, including LGR5.

## References

[1] Ferlay J, Soerjomataram I, Dikshit R, Eser S, Mathers C, Rebelo M, Parkin DM, Forman D, Bray F (2015). Cancer incidence and mortality worldwide: sources, methods and major patterns in GLOBOCAN 2012. International journal of cancer.

[2] Torre LA, Bray F, Siegel RL, Ferlay J, Lortet-Tieulent J, Jemal A (2015). Global cancer statistics, 2012. CA: a cancer journal for clinicians.

[3] Correa P (2013). Gastric cancer: overview. Gastroenterology clinics of North America.

[4] Dalessandri T, Strid J (2014). Beneficial autoimmunity at body surfaces-immune surveillance and rapid type 2 immunity regulate tissue homeostasis and cancer. Frontiers in immunology.

[5] Hoffmann W (2013). Self-renewal of the gastric epithelium from stem and progenitor cells. Frontiers in bioscience (Scholar edition).

[6] Clevers H, Loh KM, Nusse R (2014). Stem cell signaling. An integral program for tissue renewal and regeneration: Wnt signaling and stem cell control. Science.

[7] Soteriou D, Fuchs Y (2018). A matter of life and death: stem cell survival in tissue regeneration and tumour formation. Nature reviews cancer.

[8] Morrison SJ, Spradling AC (2008). Stem cells and niches: mechanisms that promote stem cell maintenance throughout life. Cell.

[9] Fouad YA, Aanei C (2017). Revisiting the hallmarks of cancer. American journal of cancer research.

[10] Youssef KK, Van Keymeulen A, Lapouge G, Beck B, Michaux C, Achouri Y, Sotiropoulou PA, Blanpain C (2010). Identification of the cell lineage at the origin of basal cell carcinoma. Nature cell biology.

[11] Ge Y, Gomez NC, Adam RC, Nikolova M, Yang H, Verma A, Lu CP, Polak L, Yuan S, Elemento O, Fuchs E (2017). Stem Cell lineage infidelity drives wound repair and cancer. Cell.

[12] Byun T, Karimi M, Marsh JL, Milovanovic T, Lin F, Holcombe RF (2005). Expression of secreted Wnt antagonists in gastrointestinal tissues: potential role in stem cell homeostasis. Journal of clinical pathology.

[13] Clements WM, Wang J, Sarnaik A, Kim OJ, MacDonald J, Fenoglio-Preiser C, Groden J, Lowy AM (2002). Beta-catenin mutation is a frequent cause of Wnt pathway activation in gastric cancer. Cancer research.

[14] Cai C, Zhu X (2012). The Wnt/β-catenin pathway regulates self-renewal of cancer stem-like cells in human gastric cancer. Molecular medicine reports.

[15] Reya T, Clevers H (2005). Wnt signalling in stem cells and cancer. Nature.

[16] Fafilek B, Krausova M, Vojtechova M, Pospichalova V, Tumova L, Sloncova E, Huranova M, Stancikova J, Hlavata A, Svec J, Sedlacek R, Luksan O, Oliverius M, Voska L, Jirsa M, Paces J, Kolar M, Krivjanska M, Klimesova K, Tlaskalova-Hogenova H, Korinek V (2013). Troy, a tumor necrosis factor receptor family member, interacts with lgr5 to inhibit wnt signaling in intestinal stem cells. Gastroenterology.

[17] de Lau W, Barker N, Low TY, Koo BK, Li VSW, Teunissen H, Kujala P, Haegebarth A, Peters PJ, van de Wetering M, Stange DE, van Es JE, Guardavaccaro D, Schasfoort RBM, Mohri Y, Nishimori K, Mohammed S, Heck AJ, Clevers H (2011). Lgr5 homologues associate with Wnt receptors and mediate R-spondin signalling. Nature.

[18] Barker N, van Es JH, Kuipers J, Kujala P, van den Born M, Cozijnsen M, Haegebarth A, Korving J, Begthel H, Peters PJ, Clevers H (2007). Identification of stem cells in small intestine and colon by marker gene Lgr5. Nature.

[19] Becker L, Huang Q, Mashimo H (2008). Immunostaining of Lgr5, an intestinal stem cell marker, in normal and premalignant human gastrointestinal tissue. The scientific world journal.

[20] Yamamoto Y, Sakamoto M, Fujii G, Tsuiji H, Kenetaka K, Asaka M, Hirohashi S (2003). Overexpression of orphan G-protein-coupled receptor, Gpr49, in human hepatocellular carcinomas with beta-catenin mutations. Hepatology (Baltimore, Md).

[21] Takahashi H, Ishii H, Nishida N, Takemasa I, Mizushima T, Ikeda M, Yokobori T, Mimori K, Yamamoto H, Sekimoto M, Doki Y, Mori M (2011). Significance of Lgr5(+ve) cancer stem cells in the colon and rectum. Annals of surgical oncology.

[22] Becker L, Huang Q, Mashimo H (2010). Lgr5, an intestinal stem cell marker, is abnormally expressed in Barrett's esophagus and esophageal adenocarcinoma. Diseases of the esophagus.

[23] Schön S, Flierman I, Ofner A, Stahringer A, Holdt LM, Kolligs FT, Herbst A (2014). Beta-catenin regulates NF-kappaB activity via TNFRSF19 in colorectal cancer cells. International journal of cancer.

[24] Spanjaard RA, Whren KM, Graves C, Bhawan J (2007). Tumor necrosis factor receptor superfamily member TROY is a novel melanoma biomarker and potential therapeutic target. International journal of cancer.

[25] Paulino VM, Yang Z, Kloss J, Ennis MJ, Armstrong BA, Loftus JC, Tran NL (2008). TROY (TNFRSF19) is overexpressed in advanced glial tumors and promotes glioblastoma cell invasion via Pyk2-Rac1 signaling. Molecular cancer research.

[26] Zidi IT, M'Farrej S M, Bergaoui SD, Ghariani NZ, Bartegi AB, Ben Amor NN, Nouira RB (2011). Tumor necrosis factor-receptor 2 and TROY gene expression patterns in cutaneous squamous cell carcinoma in a Tunisian population. Saudi medical journal.

[27] Nishioka M, Suehiro Y, Sakai K, Matsumoto T, Okayama N, Mizuno H, Ueno K, Suzuki N, Hashimoto S, Takami T, Hazama S, Nagano H, Sakaida I, Yamasaki T (2018). TROY is a promising prognostic biomarker in patients with colorectal cancer. Oncology letters.

[28] Wilhelm F, Böger C, Krüger S, Behrens HM, Röcken C (2017). Troy is expressed in human stomach mucosa and a novel putative prognostic marker of intestinal type gastric cancer. Oncotarget.

[29] Mohammadi M, Talebkhan Y, Khalili G, Mahboudi F, Massarrat S, Zamaninia L, Oghalaei A (2008). Advantage of using a home-made ELISA kit for detection of Helicobacter pylori infection over commercially imported kits. Indian journal of medical microbiology.

[30] Kitahara F, Kobayashi K, Sato T, Kojima Y, Araki T, Fujino MA (1999). Accuracy of screening for gastric cancer using serum pepsinogen concentrations. Gut.

[31] Eybpoosh S, Talebkhan Y, Saberi S, Esmaeili M, Oghalaie A, Ebrahimzadeh F, Karimi T, Abdirad A, Nahvijou A, Mohagheghi MA, Eshagh Hosseini M, Mohammadi M (2015). Age-specific gastric cancer risk indicated by the combination of Helicobacter pylori sero-status and serum pepsinogen levels. Iranian biomedical journal.

[32] Martin IG, Dixon MF, Sue-Ling H, Axon AT, Johnston D (1994). Goseki histological grading of gastric cancer is an important predictor of outcome. Gut.

[33] Sobin LH, Fleming ID (1997). TNM classification of malignant tumors, fifth edition (1997). Union Internationale Contre le Cancer and the American Joint Committee on Cancer. Cancer.

[34] Douraghi M, Kashani SS, Zeraati H, Esmaili M, Oghalaie A, Mohammadi M (2010). Comparative evaluation of three supplements for Helicobacter pylori growth in liquid culture. Current microbiology.

[35] Nozaki K, Shimizu N, Inada K, Tsukamoto T, Inoue M, Kumagai T, Sugiyama A, Mizoshita T, Kaminishi M, Tatematsu M (2002). Synergistic promoting effects of Helicobacter pylori infection and high-salt diet on gastric carcinogenesis in Mongolian gerbils. Japanese journal of cancer research.

[36] Zheng ZX, Sun Y, Bu ZD, Zhang LH, Li ZY, Wu AW, Wu XJ, Wang XH, Cheng XJ, Xing XF, Du H, Ji JF (2013). Intestinal stem cell marker LGR5 expression during gastric carcinogenesis. World journal of gastro-enterology.

[37] Simon E, Petke D, Boger C, Behrens HM, Warneke V, Ebert M, Röcken C (2012). The spatial distribution of LGR5+ cells correlates with gastric cancer progression. PLoS one.

[38] Bu Z, Zheng Z, Zhang L, Li Z, Sun Y, Dong B, Wu A, Wu X, Wang X, Cheng X, Xing X, Li Y, Du H, Ji J (2013). LGR5 is a promising biomarker for patients with stage I and II gastric cancer. Chinese journal of cancer research.

[39] Zhou L, Yu L, Feng ZZ, Gong XM, Cheng ZN, Yao N, Wang DN, Wu SW (2015). Aberrant expression of markers of cancer stem cells in gastric adenocarcinoma and their relationship to vasculogenic mimicry. Asian Pacific journal of cancer prevention.

[40] La Vecchia C, Negri E, Franceschi S, Gentile A (1992). Family history and the risk of stomach and colorectal cancer. Cancer.

[41] Yusefi AR, Bagheri Lankarani K, Bastani P, Radinmanesh M, Kavosi Z (2018). Risk factors for gastric cancer: A systematic review. Asian Pacific journal of cancer prevention.

[42] Uehara T, Ma D, Yao Y, Lynch JP, Morales K, Ziober A, Feldman M, Ota H, Sepulveda AR H (2013). pylori infection is associated with DNA damage of Lgr5-positive epithelial stem cells in the stomach of patients with gastric cancer. Digestive diseases and sciences.

[43] Choi YJ, Kim N, Lee HS, Park SM, Park JH, Yoon H, Shin CM, Park YS, Kim JW, Lee DH (2016). Expression of leucine-rich repeat-containing G-protein coupled receptor 5 and CD44: potential implications for gastric cancer stem cell marker. Journal of cancer prevention.

[44] Miki K, Ichinose M, Kakei N, Yahagi N, Matsushima M, Tsukada S, Ishihama S, Shimizu Y, Suzuki T, Kurokawa K, Takahashi K (1995). The clinical application of the serum pepsinogen I and II levels as a mass screening method for gastric cancer. Advances in experimental medicine and biology.

[45] Watabe H, Mitsushima T, Yamaji Y, Okamoto M, Wada R, Kokubo T, Doi H, Yoshida H, Kawabe T, Omata M (2005). Predicting the development of gastric cancer from combining Helicobacter pylori antibodies and serum pepsinogen status: a prospective endoscopic cohort study. Gut.

[46] Dinis-Ribeiro M, Yamaki G, Miki K, Costa-Pereira A, Matsukawa M, Kurihara M (2004). Meta-analysis on the validity of pepsinogen test for gastric carcinoma, dysplasia or chronic atrophic gastritis screening. Journal of medical screening.

[47] Wu C, Xie Y, Gao F, Wang Y, Guo Y, Tian H, Li Y, Fan W (2013). Lgr5 expression as stem cell marker in human gastric gland and its relatedness with other putative cancer stem cell markers. Gene.

[48] Xi HQ, Cai AZ, Wu XS, Cui JX, Shen WS, Bian SB, Wang N, Li JY, Lu CR, Song Z, Wei B, Chen L (2014). Leucine-rich repeat-containing G-protein-coupled receptor 5 is associated with invasion, metastasis, and could be a potential therapeutic target in human gastric cancer. British journal of cancer.

[49] Kalantari E, Asadi Lari MH, Roudi R, Korourian A, Madjd Z (2017). Lgr5High/DCLK1High phenotype is more common in early stage and intestinal subtypes of gastric carcinomas. Cancer biomarkers.

[50] Xi HQ, Cui JX, Shen WS, Wu XS, Bian SB, Li JY, Song Z, Wei B, Chen L (2014). Increased expression of Lgr5 is associated with chemotherapy resistance in human gastric cancer. Oncology reports.

[51] Chen XL, Chen XZ, Wang YG, He D, Lu ZH, Liu K, Zhang WH, Wang W, Li CC, Xue L, Zhao LY, Yang K, Liu JP, Zhou ZG, Hu JK, Mo XM (2016). Clinical significance of putative markers of cancer stem cells in gastric cancer: A retrospective cohort study. Oncotarget.

[52] Sigal M, Rothenberg ME, Logan CY, Lee JY, Honaker RW, Cooper RL, Passarelli B, Camorlinga M, Bouley DM, Alvarez G, Nusse R, Torres J, Amieva MR (2015). Helicobacter pylori activates and expands Lgr5(+) stem cells through direct colonization of the gastric glands. Gastroenterology.

[53] Oh JD, Karam SM, Gordon JI (2005). Intracellular Helicobacter pylori in gastric epithelial progenitors. Proceedings of the national academy of sciences of the United States of America.

[54] Buti L, Spooner E, Van der Veen AG, Rappuoli R, Covacci A, Ploegh HL (2011). Helicobacter pylori cytotoxin-associated gene A (CagA) subverts the apoptosis-stimulating protein of p53 (ASPP2) tumor suppressor pathway of the host. Proceedings of the national academy of sciences of the United States of America.

[55] Amieva MR, Vogelmann R, Covacci A, Tompkins LS, Nelson WJ, Falkow S (2003). Disruption of the epithelial apical-junctional complex by Helicobacter pylori CagA. Science.

[56] Higashi H, Tsutsumi R, Muto S, Sugiyama T, Azuma T, Asaka M, Hatakeyama M (2002). SHP-2 tyrosine phosphatase as an intracellular target of Helicobacter pylori CagA protein. Science.

[57] Wu H, Whitfield TW, Gordon JA, Dobson JR, Tai PW, van Wijnen AJ, Stein JL, Stein GS, Lian JB (2014). Genomic occupancy of Runx2 with global expression profiling identifies a novel dimension to control of osteoblastogenesis. Genome biology.

[58] Qiu W, Hu Y, Andersen TE, Jafari A, Li N, Chen W, Kassem M (2010). Tumor necrosis factor receptor superfamily member 19 (TNFRSF19) regulates differentiation fate of human mesenchymal (stromal) stem cells through canonical Wnt signaling and C/EBP. The journal of biological chemistry.

[59] Qiao XT, Gumucio DL (2011). Current molecular markers for gastric progenitor cells and gastric cancer stem cells. Journal of gastroenterology.

[60] Walker R, Poleszczuk J, Mejia J, Lee JK, Pimiento JM, Malafa M, Giuliano AR, Enderling H, Coppola D (2018). Toward early detection of Helicobacter pylori-associated gastric cancer. Gastric cancer.

[61] Walker R, Mejia J, Lee JK, Pimiento JM, Malafa M, Giuliano AR, Coppola D, Enderling H (2017). Personalizing gastric cancer screening with predictive modeling of disease progression biomarkers. Applied immunohistochemistry and molecular morphology.

[62] Zhao H, Wen J, Dong X, He R, Gao C, Zhang W, Zhang Z, Shen L (2017). Identification of AQP3 and CD24 as biomarkers for carcinogenesis of gastric intestinal metaplasia. Oncotarget.

[63] Levi E, Sochacki P, Khoury N, Patel BB, Majumdar AP (2014). Cancer stem cells in Helicobacter pylori infection and aging: Implications for gastric carcinogenesis. World journal of gastrointestinal pathophysiology.

